# In response: Letter on update to the Vitamin C, Thiamine and Steroids in Sepsis (VICTAS) protocol

**DOI:** 10.1186/s13063-020-04290-6

**Published:** 2020-04-22

**Authors:** Christopher J. Lindsell, Anna McGlothlin, Samuel Nwosu, Todd W. Rice, Alex Hall, Gordon R. Bernard, Laurence W. Busse, E. Wesley Ely, Alpha A. Fowler, David F. Gaieski, Jeremiah S. Hinson, Michael H. Hooper, James C. Jackson, Gabor D. Kelen, Mark Levine, Greg S. Martin, Richard E. Rothman, Jonathan E. Sevransky, Kert Viele, David W. Wright, David N. Hager

**Affiliations:** 1 Department of Biostatistics, Vanderbilt University Medical Center, Nashville, TN USA; 2 Berry Consultants, LLC, Austin, TX USA; 3 Division of Pulmonary & Critical Care Medicine, Department of Medicine, Vanderbilt University School of Medicine, Nashville, TN USA; 4 Department of Emergency Medicine, Emory University, Atlanta, GA USA; 5 Grady Memorial Hospital, Atlanta, GA USA; 6 Division of Pulmonary, Allergy, Critical Care, and Sleep Medicine, Department of Medicine, Emory University, Atlanta, GA USA; 7 Division of Pulmonary & Critical Care, Department of Medicine, Vanderbilt University Medical Center, Nashville, TN USA; 8 Critical Illness, Brain Dysfunction, and Survivorship (CIBS) Center, Vanderbilt University Medical Center, Nashville, TN USA; 9 Tennessee Valley Veteran’s Affairs Geriatric Research Education Clinical Center (GRECC), Nashville, TN USA; 10 Division of Pulmonary Disease & Critical Care Medicine, Department of Internal Medicine, The VCU Johnson Center for Critical Care and Pulmonary Research, Virginia Commonwealth University School of Medicine, Richmond, VA USA; 11 Department of Emergency Medicine, Sidney Kimmel Medical College, Thomas Jefferson University, Philadelphia, PA USA; 12 Department of Emergency Medicine, Johns Hopkins University, Baltimore, MD USA; 13 Division of Pulmonary & Critical Care Medicine, Department of Medicine, Eastern Virginia Medical School and Sentara Healthcare, Norfolk, VA USA; 14 Critical Illness, Brain Dysfunction, and Survivorship (CIBS) Center, Vanderbilt University Medical Center, Nashville, TN USA; 15 Tennessee Valley Veteran’s Affairs Geriatric Research Education Clinical Center (GRECC), Nashville, TN USA; 16 Department of Psychiatry, Vanderbilt University School of Medicine, Nashville, TN USA; 17 Molecular & Clinical Nutrition Section, Intramural Research Program, National Institute of Diabetes and Digestive and Kidney Diseases, National Institutes of Health, 10 Center Drive, Bethesda, MD USA; 18 Division of Pulmonary, Allergy, Critical Care, and Sleep Medicine, Department of Medicine, Emory University, Emory Critical Care Center, Atlanta, GA USA; 19 Division of Pulmonary & Critical Care Medicine, Department of Medicine, Johns Hopkins University, 1800 Orleans Street, Suite 9121, Baltimore, MD 21287 USA

## Abstract

ClinicalTrials.gov: NCT03509350. Registered on 26 April 2018.

To the Editors,

We thank Drs. Frommelt, Kory, and Long for their interest in the Vitamin C, Thiamine and Steroids in Sepsis (VICTAS) trial and their recommendation to carefully consider time to treatment in our analysis plan. As they have pointed out, our inclusion criteria require study drug administration within 28 h of the onset of first qualifying organ dysfunction [1]. While it is unknown what differential effect time to hydrocortisone, ascorbic acid, and thiamine (commonly referred to as HAT therapy) may have on patients with sepsis, we agree early treatment with other therapeutics has improved outcomes in sepsis [2]. However, in planning the VICTAS trial, we chose to allow a moderately wide enrollment window reasoning that HAT therapy, if effective, would be of interest to providers managing patients along a wide spectrum of illness severity.

Since completing enrollment in the VICTAS trial in late 2019, discussions of trials exploring the benefits of therapeutic regimens like the one we tested have broadened. While much focus at the time VICTAS was designed was on the question of the efficacy of HAT, negative trials have stimulated a discussion as to when HAT therapy could be effective [3, 4].

In response, we have calculated the time between first qualifying organ dysfunction and first dose of study intervention in the VICTAS trial. This will be reported along with other participant characteristics and will be included in our adjusted analyses following the same approach as described for other covariates. Specifically, we will use restricted cubic splines to address potential non-linearities, and we will assess for a differential treatment effect. This will be done by testing the interaction between time to treatment and treatment assignment. If the interaction achieves a *P* value ≤ 0.2, we will consider the possibility of subgroup analyses with grouping informed by the relationship between time to treatment and outcomes. Since the approach was specified a priori, this helps to mitigate the post hoc addition of this covariate. We expect that including this important variable will strengthen our findings given the suggestion that time to treatment may modify the response to treatment.

## Data Availability

A de-identified dataset from participants in the VICTAS trial will be made publicly available approximately 1 year after publication of the primary manuscript.

## Abbreviations

HAT: Hydrocortisone, ascorbic acid, and thiamine
VICTAS: Vitamin C, Thiamine and Steroids in Sepsis

## References

[1] Hager DN, Hooper MH, Bernard GR, Busse LW, Ely EW, Fowler AA (2019). The Vitamin C, Thiamine and Steroids in Sepsis (VICTAS) Protocol: a prospective, multi-center, double-blind, adaptive sample size, randomized, placebo-controlled, clinical trial. Trials.

[2] Kumar A, Roberts D, Wood KE, Light B, Parrillo JE, Sharma S (2006). Duration of hypotension before initiation of effective antimicrobial therapy is the critical determinant of survival in human septic shock. Crit Care Med.

[3] Marik PE, Payen D (2019). CITRIS-ALI: How statistics were used to obfuscate the true findings. Anaesth Crit Care Pa.

[4] Brant EB, Angus DC (2019). Is high-dose vitamin C beneficial for patients with sepsis?. JAMA.

